# Unveiling Vaccine Hesitancy on Twitter: Analyzing Trends and Reasons during the Emergence of COVID-19 Delta and Omicron Variants

**DOI:** 10.3390/vaccines11081381

**Published:** 2023-08-18

**Authors:** Liviu-Adrian Cotfas, Liliana Crăciun, Camelia Delcea, Margareta Stela Florescu, Erik-Robert Kovacs, Anca Gabriela Molănescu, Mihai Orzan

**Affiliations:** 1 Department of Economic Informatics and Cybernetics, Bucharest University of Economic Studies, 010374 Bucharest, Romania; 2 Department of Economics and Economic Policies, Bucharest University of Economic Studies, 010374 Bucharest, Romania; 3 Department of Administration and Public Management, Bucharest University of Economic Studies, 010374 Bucharest, Romania; 4 Department of Marketing, Bucharest University of Economic Studies, 010374 Bucharest, Romania

**Keywords:** opinion mining, social media, COVID-19, SARS-CoV-2, stance classification, vaccine

## Abstract

Given the high amount of information available on social media, the paper explores the degree of vaccine hesitancy expressed in English tweets posted worldwide during two different one-month periods of time following the announcement regarding the discovery of new and highly contagious variants of COVID-19—Delta and Omicron. A total of 5,305,802 COVID-19 vaccine-related tweets have been extracted and analyzed using a transformer-based language model in order to detect tweets expressing vaccine hesitancy. The reasons behind vaccine hesitancy have been analyzed using a Latent Dirichlet Allocation approach. A comparison in terms of number of tweets and discussion topics is provided between the considered periods with the purpose of observing the differences both in quantity of tweets and the discussed discussion topics. Based on the extracted data, an increase in the proportion of hesitant tweets has been observed, from 4.31% during the period in which the Delta variant occurred to 11.22% in the Omicron case, accompanied by a diminishing in the number of reasons for not taking the vaccine, which calls into question the efficiency of the vaccination information campaigns. Considering the proposed approach, proper real-time monitoring can be conducted to better observe the evolution of the hesitant tweets and the COVID-19 vaccine hesitation reasons, allowing the decision-makers to conduct more appropriate information campaigns that better address the COVID-19 vaccine hesitancy.

## 1. Introduction

Since the announcement made on 9 November 2020 regarding the production of a vaccine that has a 90% efficiency rate against the novel coronavirus SARS-CoV-2, people worldwide have started to express their opinions related to their decisions to take the vaccine against the COVID-19 disease or not [1,2]. Among the most well-known social media platforms, Twitter has been preferred by users from different countries as it offers the possibility to write relative short text messages, accompanied or not by pictures and links, in which users can simply express their point of view regarding different aspects of their everyday lives [3]. As a result, the ease of creating and sharing content offered by the platform has enabled the amplification of rumors and questionable information during the COVID-19 pandemic [4], which has increased the interest of the scientific community to deeply analyze the tweets in order to extract information related to the evolution of the COVID-19 pandemic and people’s opinions [5,6,7]. 

Based on the studies published by the scientific community, it has been observed that people’s COVID-19 vaccine attitudes vary considerably based on their country of residence and the socio-demographic characteristics of the people taking part in the surveys [8]. Regarding the country-residency, it has been determined that the COVID-19 vaccine acceptance rate ranges between 35.3% and 94.1% for the countries in the European Union [9,10,11], 50.0% and 96.7% in the United States [12,13,14,15,16], 68.7% and 80.0% in Canada [15,17], 76.5% and 86% in Australia [18,19], 34.8% and 88.6% in China [17,20], 45.0% and 54.9% in Russia [17,21], 51.5% and 65.2% in Nigeria [17,22]. As for the socio-demographic characteristics, vaccine hesitancy has often been associated with young people, low education levels, inadequate health literacy, female gender, and some specific ethnic groups [19,23,24,25]. Additionally, it has been shown that the attitude towards the COVID-19 vaccination tends to be more hesitant than in the case of other vaccines [26] and that people with a history of vaccine refusal are more likely to refuse the COVID-19 vaccine [27]. 

The term of vaccine hesitancy can be understood in both a narrow sense—having the meaning of delaying the decision regarding vaccination even though a vaccine is available [28]—and a broad sense—comprising also the situation in which the person refuses to vaccinate even though the vaccine is available [29,30]. In the present paper, the term is used in a broad sense, following the works from the scientific literature [31,32,33,34] and the World Health Organization (WHO) reports [35], which consider that vaccine refusal is only a part of vaccine hesitancy. 

As presented above, the hesitancy towards COVID-19 vaccination has been mostly studied in the scientific literature by considering a country-related approach through surveys on relatively small data samples. The main drawbacks related to using questionnaires when analyzing people’s hesitancy towards vaccination are related to the representativeness of the selected sample in the overall population and to the fact that the opinion can be extracted only over limited periods of time [36]. Instead, by using large datasets extracted from social media, comprising people’s opinions related to COVID-19 vaccination, one can easily extract and analyze the influence of a news story or campaign over the changes in attitudes towards COVID-19 vaccination. As it has been shown in scientific research, the analysis of Twitter-related data can be useful in better understanding different changes in people’s opinions [2,37,38,39,40]. Having insight into people’s opinions towards COVID-19 vaccination can improve governance and play a crucial role in controlling the COVID-19 pandemic [41]. 

To monitor the people’s hesitancy towards COVID-19 vaccination in the one-month period following the announcement of the occurrence of Delta and Omicron variants (4 April 2021–3 May 2021 and 24 November 2021–23 December 2021, respectively), 5,305,802 tweets have been extracted and analyzed using a Transformer-based language model for the purpose of determining the hesitant tweets. The topics and reasons behind the COVID-19 vaccination hesitancy have been further analyzed using a Latent Dirichlet Allocation approach. A comparison between the COVID-19 vaccine hesitancy tweets in the two mentioned periods is provided with the purpose of determining if differences can be identified in terms of degree and reasons leading to hesitancy. Additionally, a comparison between the tweets extracted at the beginning of the vaccination process [8], the ones after the start of the administration of the third vaccine booster dose [42], and the ones in the two considered periods is provided. Following the same approach, people’s opinions related to COVID-19 vaccine hesitancy can be monitored in real time, which could facilitate the creation of more appropriate information campaigns that could better address the hesitancy reasons in a more immediate manner. 

As such, the main research questions we have considered are as follows:

RQ1: Are there differences between the two periods with regards to the proportion and trend of hesitant tweets?

RQ2: Are there differences between the two periods with regards to the number and contents of the topics discussed within hesitant tweets?

RQ3: What long-term trends can we identify if we compare the two mentioned timespans to other timespans that have been studied in the literature, such as the beginning of the vaccination process or the start of the administration of the third vaccine booster dose, from the point of view of the proportion and contents of hesitant tweets?

## 2. Literature Review 

In this section, a selection of papers is made based on their focus on Twitter as a source for data extraction in the context of COVID-19 or for discussing aspects related to stance detection.

### 2.1. Twitter as a Source of Data in COVID-19 Context

In the context of the COVID-19 pandemic, the crucial role played by social media and its potential for use in identifying the prevailing discourses among the public have long been noted [43,44,45]. For instance, under the umbrella of e-government, sentiment analysis of social media texts can enable government decision-makers to assess public opinion with regards to policies such as the vaccine rollout in order to improve their effectiveness [46].

Lanyi et al. [47] underscore the importance of targeted vaccination strategies in the case of COVID-19, focusing on groups where uptake is low and identifying reasons for vaccine hesitancy. The authors highlight the importance of AI techniques and of real-time analysis of public attitudes and discussion topics sourced from social media in the face of the difficulties and cost of traditional methods such as surveys or focus groups, especially in the context of the pandemic. The researchers demonstrated the viability of the method by retrieving a set of 91,473 tweets geo-located to London, UK, over an 8-month period from November 2020 to August 2021 and using proprietary off-the-shelf NLP software to identify tweets with negative sentiment and determine the most relevant topics [47]. Based on this clustering, the authors then use qualitative analysis of the most relevant tweets assigned to each topic to gain a more nuanced understanding of the public’s views. The researchers conclude that safety concerns, mistrust of government and healthcare authorities, accessibility of the vaccine, and complacency are the key themes expressed in their sample. The authors also observe the widespread dissemination of misinformation regarding the vaccine on social media. Interestingly enough, while misinformation is ordinarily associated with the vaccine-hesitant community [48], Lanyi et al. [47] report the presence of a small number of users sharing misinformation in support of vaccination.

Feng and Zhou [49] have studied the public’s opinions with regards to pandemic-era policies such as travel restrictions, lockdowns, and working from home using a 650,000 text-long geo-tagged dataset sourced from Twitter posted in the US between January and May 2020. The authors also highlight the difficulty of using traditional questionnaires in this context. Using this dataset, the researchers studied work engagement at the county, state, and national levels during this period based on tweet volumes. The authors use hashtags, @mentions, and a Latent Dirichlet Allocation model to determine topics of interest expressed during this period and also study their distribution across time, noting that different topics are more active at different times. The researchers then perform emoji-based sentiment analysis and event identification, highlighting the negative sentiment associated with certain pandemic milestones, such as the number of deaths [49].

Finally, Bonifazi et al. [50] use data from Twitter collected between October 2020 and April 2021 to model conversations on Twitter with a multilayer social network approach. The authors identify the stance of tweets using “gold-standard” hashtags instead of stance detection and group tweets into “pro-vaxxer”, neutral, and “anti-vaxxer” communities. The researchers conclude in their approach that anti-vaxxer networks are more densely connected and more cohesive than pro-vaxxer ones, which leads to higher interactions within these networks [50].

### 2.2. Stance Detection

Sentiment analysis is a set of techniques that can be used to infer people’s subjective views as expressed in unstructured texts [51]. Cui et al. [52] highlight that most of the text content generated on the internet in the Web 2.0 era is closely related to the subjective experiences of users and, as such, highly applicable to surveys of public opinion, known as sentiment analysis. The authors note the increasing popularity of this approach not only among researchers but also among companies and governments. The results of the analysis reveal the most frequented areas of research and the most commonly used methods [52].

In contrast to generic sentiment analysis, stance detection aims to identify the public’s opinions in relation to a concrete target, such as different issues, topics, events, products, political candidates, etc. Torregrosa et al. [53] study the political discourse on Twitter from a sentiment analysis and “campaign tone” perspective as applied to the 2021 Spanish local elections in the Autonomous Community of Madrid. The authors then perform an n-gram and hashtag analysis of the results in order to facilitate a qualitative analysis of the debate. The researchers focus on the negative tone and conclude that Twitter can be used as a “sentiment thermometer” to investigate political campaigns from different parties [53].

Finally, Martinez et al. [54] have studied the COVID-19 vaccination campaign from a stance detection perspective using an annotated corpus of Spanish-language tweets they contributed themselves. The authors mention the applications of stance detection to citizen sensing and public health applications. The researchers identified relevant topics using the clustering method on the data instead of traditional topic modeling, using SBERT sentence embeddings as well as HDBSCAN. An interesting finding they present is that they found, during the manual annotation phase, that the conventional three-class (in favor, neutral, and against) schema associated with stance detection was insufficient to capture the nuances present in the discourse [54].

## 3. Methodology

**Figure 1:**
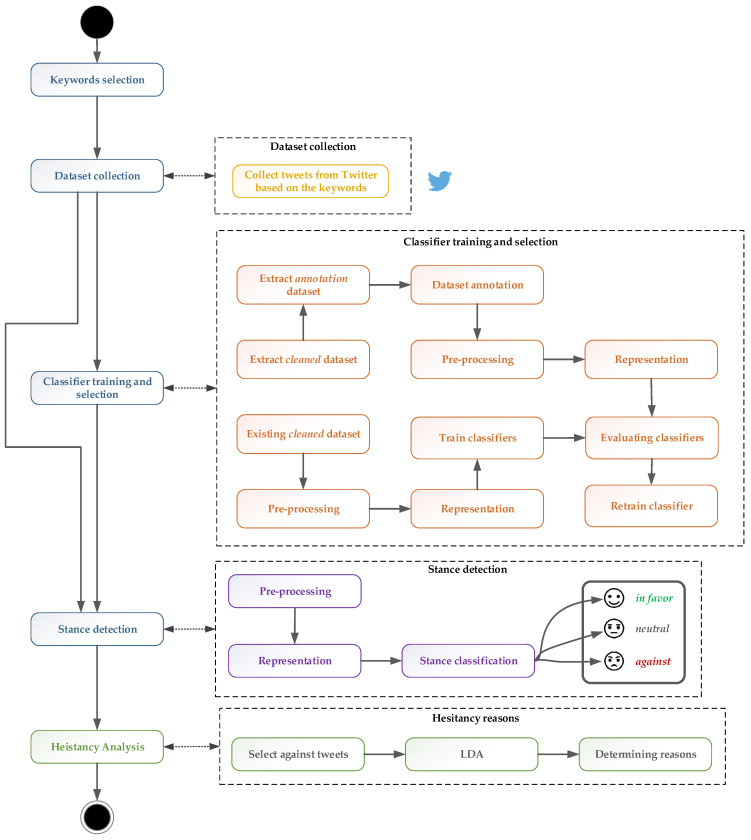
COVID-19 vaccine hesitancy analysis.

In order to extract and analyze the hesitant tweets related to COVID-19 vaccination, a series of steps are needed, as presented in Figure 1 and discussed in the following. 

We would like to state that no experiment involving human subjects has been performed in the paper. As presented below, the messages posted by users on Twitter have been extracted and analyzed using machine learning and deep learning classifiers. We have not interfered with the process by which the users have posted their opinions on Twitter, as the extraction process has been made after the tweets have been posted by the users.

### 3.1. Keyword Selection

During this step, a series of keywords related to COVID-19 and vaccination were selected based on the scientific literature. The keywords used for the tweet extraction in the case of the present study are related to both COVID-19 (covid19, covid-19, coronavirus, coronaoutbreak, coronaviruspandemic, wuhanvirus, 2019nCoV) and vaccination (vaccine, vaccination, vaccinate, vaccinating, vaccinated).

### 3.2. Dataset Collection

First, a *cleaned* dataset is created by removing from the *entire* dataset the retweets and duplicate tweets as suggested in the scientific literature [3,55]. This action ensures that the resulting dataset contains only unique user-posted tweets. Further on, a percentage of the tweets in the *cleaned* dataset is randomly selected in order to be annotated by three independent persons into three categories: *in favor*—tweets that support the COVID-19 vaccination; *neutral*—mainly represented by news and tweets not expressing a for/against attitude for COVID-19 vaccination; and *against*—tweets that reject the COVID-19 vaccination or postpone it for the moment.

The results in terms of annotation are compared between the three annotators, and, in the case of disagreement, the category mentioned by most of the annotators is selected. From the *annotated* dataset, a *balanced* dataset is extracted. This action is needed as the use of an unbalanced dataset for training could adversely affect the performance of the considered classifiers. The next step is represented by pre-processing, through which the user mentions, emails, and links are removed, the hashtags are unpacked, the emoticons are replaced by their corresponding words, the elongated words are adjusted, minor spelling mistakes are corrected, and all words are spelled using lowercase. These pre-processing actions are needed, as it has been proven that they are crucial in achieving results that are as close as possible to the users’ opinions [3]. To this extent, the ekphrasis library, the Natural Language Toolkit (NLTK) library, and the “re” Python module are used [36,56,57,58]. Next, the text is represented as numbers to ensure that it can be handled by the classification algorithms. For classical machine learning algorithms, the representation is made through the use of the n-gram language model. Additionally, for the algorithms that rely on the word’s frequency, the term frequency-inverse document frequency (TF-IDF) is investigated for increasing their performance. 

Afterwards, for the classifiers training, a relatively large *existing balanced* annotated dataset that has been extracted in previous studies based on the same keywords is used. To this set, the pre-processing and representation steps presented above are applied. Once these two steps are fulfilled, the classifiers are trained on the resulting dataset and tested on the dataset that has been annotated in this study. A series of both classical machine learning and deep learning classifiers are considered for training: Multinomial Naive Bayes (MNB) [59], Random Forest (RF) [60], Support Vector Machine (SVM) [61], Bidirectional Encoder Representations from Transformers (BERT) [62], Robustly Optimized BERT Pretraining Approach (RoBERTa) [63], and A Lite BERT (ALBERT) [64]. The performance of the considered classifiers is measured using four metrics [3]:

(1) $$\mathrm{Accuracy}=\frac{TP+TN}{TP+TN+FP+FN}$$

(2) $$\mathrm{Precision}=\frac{TP}{TP+FP}$$

(3) $$\mathrm{Recall}=\frac{TP}{TP+FN}$$

(4) $$F-\mathrm{score}=2\cdot \frac{\mathrm{Precision}\;\cdot \;\;\mathrm{Recall}}{\mathrm{Precision}+\mathrm{Recall}}$$

where *TP* is the number of real positive tweets classified as positive; *FP* is the number of real negative tweets classified incorrectly as positive; *TN* represents the number of negative tweets correctly classified as negative; and *FN* is the number of real positive tweets incorrectly classified as negative. 

Higher values of the four metrics are preferred for selecting the best-performing classifier. 

After the best-performing classifier is selected in terms of accuracy, precision, recall, and F-score, the *balanced* annotated dataset extracted for the current period and pre-processed as mentioned above is added to the *existing balanced* annotated dataset on which the classifier’s training has been performed. The resulting dataset is further used for the re-training of the best-performing classifier in order to further increase its accuracy. 

### 3.3. Stance Detection

The *entire* dataset is transformed through a pre-processing and representation step, as presented above. The tweets in the resulting dataset are classified using the best-performing classifier into three categories: *in favor*, *neutral,* and *against*. The evolution of the number of tweets in each category and their contribution to the total number of tweets are discussed and put in connection with the major events that occurred in the selected period for better observing if there is a relationship between their dynamics and the major events. In order to facilitate the discovery of the major events, an n-gram analysis is performed. From the entire dataset, the corresponding cleaned dataset is extracted and analyzed under the same considerations. 

### 3.4. Hesitancy Analysis

The *against* tweets are extracted from the *cleaned* dataset, and a Latent Dirichlet Allocation (LDA) [65] is performed. Prior to applying LDA, the tweets in the *against* dataset are lemmatized using the spaCy Python library. Gensim library [66] is used for topic discovery, while the visualization of the topics is made with the help of pyLDAvis library. The top 30 most salient terms are extracted for each topic. 

Based on the resulting topics and their most salient words [67], the reasons behind vaccine hesitancy are determined. A discussion regarding the evolution of the reasons over time by selecting similar works in the field (especially in terms of keywords and definitions associated with the reasons behind COVID-19 vaccination hesitancy) is performed to better highlight the dynamics of the hesitancy reasons over time. 

## 4. Results

The results of the COVID-19 vaccine hesitancy are discussed in the following terms: extracted datasets, selecting the best classification model for dividing the tweets among the three considered categories, evolution of the number of tweets in the considered periods, reasons for hesitancy, and evolution of the reasons over time. 

### 4.1. Extracting the Tweets

Considering the one-month period starting from the dates the World Health Organization [68] reported that Delta is a variant of interest (4 April 2021–3 May 2021) and that Omicron is a variant under monitoring (24 November 2021–23 December 2021), 5,305,802 tweets have been extracted. The keywords used for the tweets’ extraction are related to both COVID-19 (covid19, covid-19, coronavirus, coronaoutbreak, coronaviruspandemic, wuhanvirus, 2019nCoV) and vaccination (vaccine, vaccination, vaccinate, vaccinating, vaccinated).

The division of the tweets between each of the two equally long periods has shown that the number of tweets extracted in the case of the Delta variant (3,218,843 tweets, named Delta *entire* dataset in the following) is higher than the number of tweets extracted in the case of the Omicron variant (2,086,959 tweets, named Omicron *entire* dataset). 

The *entire* dataset for each of the two periods comprises all the tweets extracted using the considered keywords, namely the original tweets and their retweets. As the opinions in the scientific literature are divided among keeping or not the retweets in the analysis [3,69]—the against camp arguing that the inclusion of the retweets will make a tweet count multiple times in the overall result, while the for camp argues that each retweet represents a new opinion as only if someone agrees or supports the content of the tweet they will be tempted to retweet it—in the following, we will consider two datasets for each period included in the analysis: the *entire* dataset—comprising all the extracted tweets, and the *cleaned* dataset—which does not contain the retweets and duplicate tweets. As a result, after removing the retweets and duplicate tweets from each of the *entire* datasets, the resulting *cleaned* datasets consist of 1,010,631 tweets in the Delta *cleaned* dataset and 515,416 tweets in the Omicron *cleaned* dataset. 

### 4.2. Selecting the Best Classifier

Based on the steps presented in the Methods section, a sample of 0.10% of the *cleaned* datasets, containing 1526 tweets, has been randomly extracted and annotated into three categories: *in favor*—representing the tweets that support the need for COVID-19 vaccination, express the willingness of people to vaccinate, present a current state in which a person declared that he/she has received the COVID-19 vaccine, and/or urge other persons to take the COVID-19 vaccine; *neutral*—representing the news related to the COVID-19 vaccine or tweets that do not express a for or against opinion regarding COVID-19 vaccination (e.g., changes in the vaccination policy, important public figures who have decided to have the vaccine or not, discussions regarding the vaccine distribution process, etc.); *against*—representing the tweets in which people express their opinion related to not taking the COVID-19 vaccine, the reasons behind not taking this decision, or their hesitancy in taking the vaccine for the moment. The tweets have been annotated by three independent annotators who have good English proficiency as well as previous experience in annotating Twitter datasets. For the selected dataset, there have not been any cases of disagreement regarding the placement of the tweets in one of the three categories, except for a very few cases in which agreement has been established by choosing the category that has been selected by most annotators. These cases have appeared when choosing between *neutral* and *in favor* and *neutral* and *against* categories, but never between *in favor* and *against* categories.

As a result of the annotation process of the 1526 tweets, 164 tweets have been marked as *in favor* (10.75%), 1249 tweets have been included in the *neutral* category (81.85%), and 113 tweets have been added to the *against* category (7.40%). A *balanced* dataset has been extracted from the annotated dataset, containing 113 tweets in each category for a total of 339 tweets.

For choosing the best-performing classifier, a *balanced* dataset, consisting of 5376 tweets extracted using the same keywords for a period equal to three months (namely 9 November 2020–8 December 2020; 8 December 2020–7 January 2021; 12 July 2021–11 August 2021) [8,36,42], has been used for training.

The performance of the considered classifiers has been evaluated on the *balanced* dataset annotated for the Delta and Omicron periods. The results of the comparison among the considered classifiers in terms of precision, recall, F-score, and accuracy are presented in Table 1.

For all the algorithms mentioned in Table 1, various hyperparameters have been investigated through a grid-search approach, such as, in the case of the classical machine learning algorithms (SVM, MNB, and RF), limiting the number of features that have been considered to 1500, 2000, and 3000 values, as well as different n-gram combinations ranging between (1,1) and (1,3). Furthermore, the algorithms underwent evaluation in both scenarios: one where general stop words were retained and another where they were omitted. The compilation of stop words utilized for this purpose corresponds to the list included within the NLTK library. Regarding the corpus-specific stop words, varying document frequency thresholds (maxDF) were examined, including values of 0.5, 0.75, and 1.0. Additionally, the assessment investigated whether the application of Term Frequency (TF) or Term Frequency—Inverse Document Frequency (TFIDF) could enhance the outcomes of stance classification. Additionally, we conducted experiments involving diverse configurations for the classifiers. For the SGDClassifier, the alpha parameter, which scales the regularization term, was adjusted across different values. Various regularization terms, including “l1,” “l2,” and “elasticnet,” were tested. The loss function of the SGDClassifier was set to “hinge,” aligning with a linear Support Vector Machine (SVM) approach. Concerning the deep learning algorithms (BERT, ALBERT, and RoBERTa), we systematically adjusted parameters such as batch size, number of epochs, and learning rate. The specific values utilized for each algorithm were sourced from established recommendations within the scientific literature.

As can be observed from Table 1, the best-performing classifier is RoBERTa, which provides the best overall results for the considered indicators. Good results are also obtained for the other two transformer-based language models, BERT (both the cased and uncased versions) and ALBERT. 

Next, the 339 tweets *balanced* dataset used for testing has been added to the 5376 tweets *balanced* dataset used for training, and the resulting dataset, containing 5715 tweets, has been used to retrain the best-performing classifier, RoBERTa, in order to further increase its classification performance. 

The last step is represented by the application of the best-performing classifier on the *entire* and *cleaned* datasets for dividing the tweets into the three-mentioned categories: *against*, *neutral*, and *in favor*. The *against* category has been assimilated to the hesitant tweets as we regard the hesitancy in a large sense, comprising the situation in which a person refuses to vaccinate even though the vaccine is available [29,30]. 

### 4.3. Comparative Analysis of Delta-Omicron Datasets

The datasets containing the classified tweets are analyzed in the following way to better observe the evolution of the number of tweets in each of the three categories considered.

#### 4.3.1. Entire Datasets

**Figure 2:**
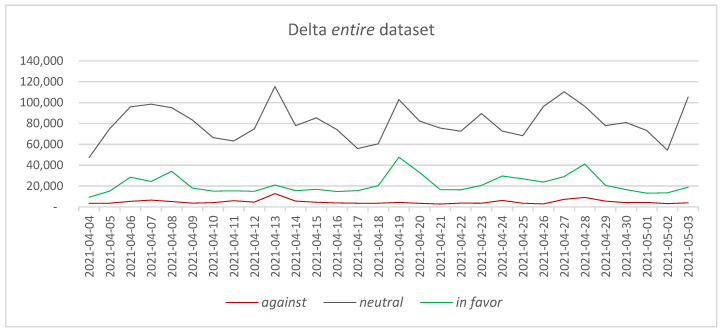
The evolution of *against*, *neutral*, and *in favor* tweets—Delta *entire* dataset.

In the case of the Delta *entire* dataset (counting for 3,218,843 tweets), it can be observed that most of the tweets are in the *neutral* category (Figure 2), representing 75.44% of the total number of extracted tweets, followed by the *in favor* tweets, accounting for 20.09% of the tweets in the dataset, and the *against* category, accounting for 4.46% of the tweets. 

Based on the daily evolution of the number of tweets in each category depicted in Figure 2, it can be observed that this hierarchy is maintained for the entire period, but at different levels. A series of peaks can be observed during the analyzed period, especially in the number of *neutral* tweets. Considering the evolution of the number of *in favor* tweets, it can be observed that it follows, in general, at a smaller scale the evolution of the number of *neutral* tweets, presenting a series of peaks almost in the same periods of time (e.g., 13 April 2021–115,500 *neutral* tweets, 21,033 *in favor* tweets, 12,761 *against* tweets; 19 April 2021–102,928 *neutral* tweets, 47,646 *in favor* tweets, 4257 *against* tweets; 3 May 2021–105,307 *neutral* tweets, 18,887 *in favor* tweets, 3879 *against* tweets). In certain situations, looking more closely at the data, one can observe a delay of one day in the increase of the number of tweets marked as *in favor* or *against* when compared with the date on which the peak in *neutral* tweets is encountered. For example, on 27 April 2021, the number of *neutral* tweets reached a peak with 110,435 tweets. On the same date, the number of *in favor* tweets was 29,076, higher than on the previous day, when 23,731 tweets were recorded, but smaller than on the next day, when the number of *in favor* tweets was 41,162. The same observation applies to the *against* tweets, whose number increased the next day, 28 April 2021. A similar situation was encountered on 7 April 2021, for both *in favor* and *against* tweets. The occurrence of these situations is normal and has been signaled in the scientific literature before, as there are cases in which the persons posting *in favor* or *against* tweets need some time in order to document or express an opinion and express it freely on Twitter [3,36]. 

Looking at the events associated with the four major peaks in the considered month, it can be observed that some major events have marked the dates on which the neutral tweets have been close to or have exceeded 100,000 tweets per day. In order to discover the events that have triggered the increase in the number of tweets, an n-gram analysis has been performed, followed by a search for the corresponding news on the Google News platform. The following events have been discovered this way:

- 7 April 2021: Medicines and Healthcare Products Regulatory Agency announces that there might be a possible link between COVID-19 vaccine AstraZeneca and extremely rare, unlikely to occur blood clots [70]—supporting n-grams: “uk government” (1380 times), “side effects” (326 times), “astrazeneca vaccine” (281 times), “experts warn” (166 times), “medical experts” (164 times), “blood clots” (98), “people got” (88 times).
- 13 April 2021: The Centers for Disease Control and Prevention and the Food & Drug Administration have released a joint statement pausing the administration of the Johnson & Johnson vaccine due to extremely rare cases of blood clots [71]—supporting n-grams: “johnson & johnson” (315 times), “side effects” (153 times), “pause johnson & johnson” (80 times), “cdc fda” (64 times), “vaccine blood clot” (56 times).
- 19 April 2021: All US adults become eligible to receive COVID-19 vaccines [72]—supporting n-grams: “side effects” (197 times), “mass vaccination” (194 times).
- 27 April 2021: A Miami private school warns that it will no longer hire teachers who get the COVID-19 vaccine [73]—supporting n-grams: “miami private school” (59 times), “employ anyone receiving covid” (18 times).
- 3 May 2021: An expert expresses worries that vaccine side effects are not sufficiently monitored [74]—supporting n-grams: “potential risks” (774 times), “side effect” (113 times).

The connection observed in the extracted dataset between the n-grams and news has also been identified in the literature when considering other periods and various vaccination themes [3,36].

**Figure 3:**
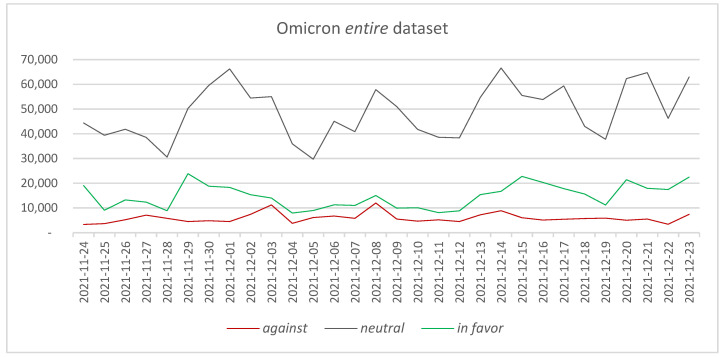
The evolution of *against*, *neutral,* and *in favor* tweets—Omicron *entire* dataset.

In the case of the Omicron *entire* dataset (counting 2,086,959 tweets), it can be observed that the same hierarchy in the number of posted tweets among the three considered categories is maintained. The *neutral* category represents 70.26% of the tweets, followed by *in favor* tweets with 21.24% and *against* tweets with 8.51%. Considering the every-day posted tweets, a similar situation as in the case of the Delta *entire* dataset is observed, namely that there are a series of peaks in the number of *neutral* tweets, which have produced some spikes in the *in favor* and *against* category—Figure 3. 

Four major peaks have been determined in the data, which have been associated through an n-gram analysis and a search on Google News platform with the following major events:

- 1 December 2021: News regarding the introduction of vaccine passports [75]—supporting n-grams: “passports” (462 times), “vaccine passports” (378 times), “passports impact” (328 times).
- 8 December 2021: Pfizer CEO declares that a fourth vaccine dose could be needed sooner than initially expected [76]—supporting n-grams: “pfizer” (7087 times), “pfizer covid” (3424 times), “double vaccinated” (2904 times), “funds pfizer” (2851).
- 14 December 2021: NHS introduces vaccination passes for children between 12 and 15 years old [77]—supporting n-grams: “children” (1026), “vaccination children” (882), “vaccination children unforgivable” (881 times).
- 21 December 2021: Sarah Palin, former Alaska governor, opposes vaccination [78]—supporting n-grams: “sarah palin” (663 time), “dead body” (660 time); “sarah palin getting vaccinated” (580 times).

Comparing the two datasets, it can be observed that the number of tweets is higher in the Delta *entire* dataset than in the Omicron *entire* dataset. The difference in the number of tweets can be due to the increased interest of the general public in the vaccination process or to the increased number of news stories associated with the occurrence of a new COVID-19 variant. Considering the data associated with the number of daily COVID-19 cases in each of the two periods, it can be observed that the number of cases in the 24 November–23 December 2021 period is higher than in the 4 April–3 May 2021 period [79]. Thus, the number of COVID-19 cases cannot be directly connected to the number of tweets posted when comparing the two periods. In terms of *in favor* tweets, it can be observed that the percentages are similar in the two periods considered: 20.09% versus 21.24%. Differences are encountered in the *against* tweets, where it can be observed that the percentage of the tweets almost doubled from the Delta variant to the Omicron variant (4.46% compared with 8.51%). Given this difference, it is interesting to determine what the main reasons are that have determined this increase between the two periods.

#### 4.3.2. Cleaned Datasets

**Figure 4:**
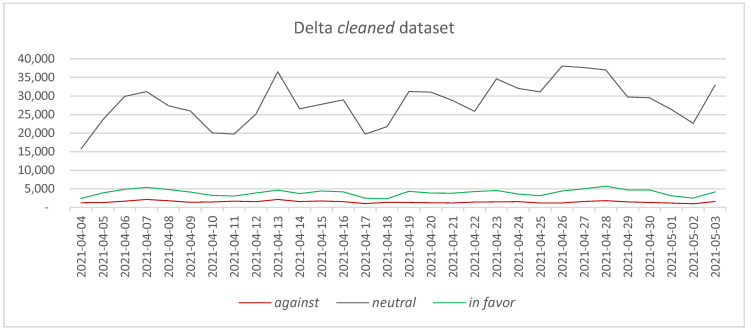
The evolution of *against*, *neutral,* and *in favor* tweets—Delta *cleaned* dataset.

**Figure 5:**
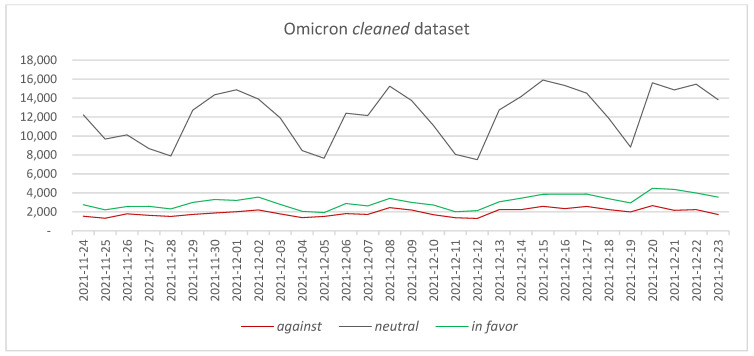
The evolution of *against*, *neutral,* and *in favor* tweets—Omicron *cleaned* dataset.

Two *cleaned* datasets have been obtained, one for each period: the Delta *cleaned* dataset, accounting for 1,010,631 tweets (Figure 4), and the Omicron *cleaned* dataset, counting for 515,416 tweets (Figure 5). Similar to the case of the *entire* datasets, a lower number of tweets can be observed for the second period, even in the case of the *cleaned* datasets. Several peaks can be identified in both *cleaned* datasets, which follow at a smaller scale the peaks in the *entire* datasets. 

In terms of types of tweets, the percentage of *neutral* tweets is 83.93% in the Delta *cleaned* dataset and 70.98% in the Omicron *cleaned* dataset. Large differences can be observed in the percentages recorded for the *against* and *in favor* tweets when comparing the two datasets. Thus, the *in favor* percentage has increased from 11.76% in the Delta *cleaned* dataset to 17.80% in the Omicron *cleaned* dataset, while the percentage of *against* tweets has increased from 4.31% in the Delta *cleaned* dataset to 11.22% in the Omicron *cleaned* dataset. Once again, the difference in the percentage of *against* tweets in the two datasets signals the need for identifying the main causes that have determined such an increase.

### 4.4. Comparative Analysis of Delta-Omicron Hesitancy Tweets

A comparative analysis, with a focus on the hesitant tweets, is discussed below for each *entire* and *cleaned* dataset. 

#### 4.4.1. Entire Datasets

**Figure 6:**
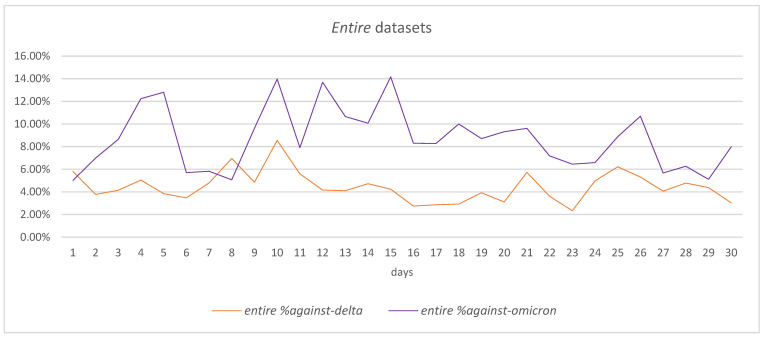
Thirty-day period comparison in *entire* datasets for *against* tweets.

Comparing the *entire* datasets in the two periods in terms of *against* tweets, it can be observed that, overall, the percentage of *against* tweets on a daily basis has been higher in the case of the Omicron *entire* dataset than in the case of the Delta *entire* dataset, with an average of 4.47% for Delta versus 8.71% for Omicron—Figure 6. 

Considering the start of each period (day 1), it can be observed that the percentage of *against* tweets is similar in the two datasets: 5.80% in the case of Delta and 5.02% in the case of Omicron. With all these, a 5-day period analysis shows that the evolution of the percentage of *against* tweets is different in the case of the two datasets: while for the Delta dataset, the percentage of *against* tweets is decreasing, reaching 3.85% in day 5, in the case of Omicron, the percentage is increasing to 12.80%. Various peaks are recorded during the 30-day period for both datasets in connection to the events that have occurred in this period, leading to an increased number of *against* tweets for the Omicron *entire* dataset in day-30 (7.98%) compared with the Delta *entire* dataset (3.03%).

#### 4.4.2. Cleaned Datasets

**Figure 7:**
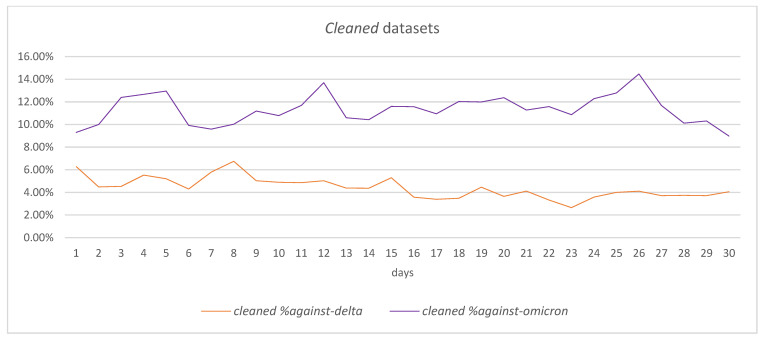
Thirty-day period comparison in *cleaned* datasets for *against* tweets.

As for the *cleaned* datasets (Figure 7), the changes in the percentage of *against* tweets are smoother across the 30-day period, even though some peaks can be observed as well. The average percentage of *against* tweets is smaller in the case of the Delta cleaned dataset (4.41%) than in the case of the Omicron cleaned dataset (11.33%).

The increasing tendency in the percentage of *against* tweets in the first 5-day period is noticeable for the Omicron *cleaned* dataset—the percentage increases from 9.30% in day 1 to 12.95% in day 5. Also, the reduction tendency in the percentage of *against* tweets in the first 5-day period for the Delta *cleaned* dataset can be observed as the percentage diminished from 6.28% in day 1 to 5.20% in day 5.

The end of the considered period, day 30, maintains the general tendency of having a higher percentage of *against* tweets in the Omicron *cleaned* dataset than in the Delta *cleaned* dataset, with 4.05% versus 8.97%.

Given the differences in the percentages for the *against* tweets in the two periods, in the following, the content of the hesitant tweets is analyzed in more depth in order to extract the reasons for people’s refusal to take the vaccine.

### 4.5. Hesitancy Analysis

A Latent Dirichlet Allocation (LDA) approach has been applied to the *cleaned* datasets to extract the reasons behind the COVID-19 vaccination hesitancy. 

#### 4.5.1. Latent Dirichlet Allocation

**Figure 8:**
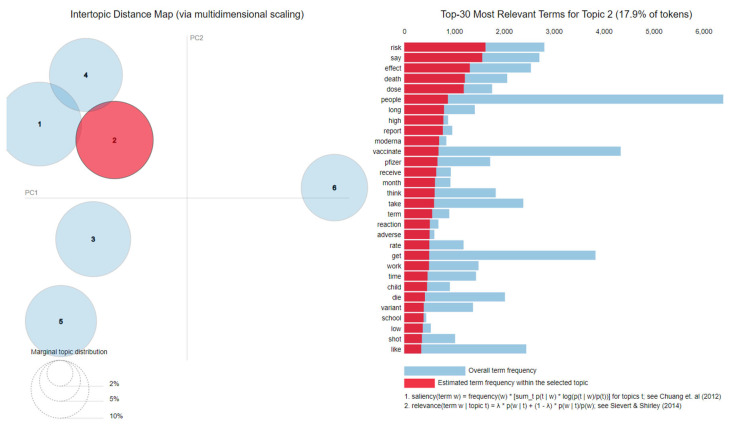
Latent Dirichlet Allocation topics and salient words in the case of Delta *cleaned* dataset [80,81].

**Figure 9:**
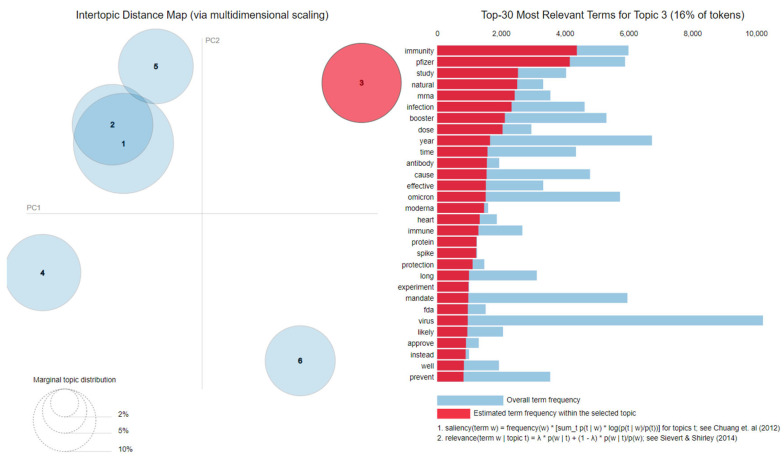
Latent Dirichlet Allocation topics and salient words in the case of Omicron *cleaned* dataset [80,81].

The results for each of the two datasets in terms of topics and the top 30 most salient terms are presented in Figure 8 and Figure 9. 

Based on the LDA analysis, the following topics have been found in the Delta *cleaned* dataset based on the identified keywords:

- Topic 1—*Side effects*: die, spread, immunity, people, protect, fully, experimental, population;
- Topic 2—*Side effects*: risk, effect, death, long, term, reaction, adverse, die;
- Topic 3—*Scam* and *Freedom*: passport, right, government, monkey, company, money, mandate;
- Topic 4—*Existence of alternatives*: prevent, mask, antibody, wear, drug, risk;
- Topic 5—*Mistrust*: guinea, pig, death, million, die, rate;
- Topic 6—*Scam*, *Hiding relevant information*, and *Side effects*: blood, clot, control, ccp, bombshell, ccpvirus, takedowntheccp, mile, guo, warn, getterantidote, medical, study.

As for the Omicron *cleaned* dataset, the identified topics have been

- Topic 1—*Side effects*: die, death, kill, body, protect, people;
- Topic 2—*Side effects*: myocarditis, blood, clot, die, death, people, cause, stop, rate, injury;
- Topic 3—*Existence of alternatives*: immunity, natural, infection, antibody, effective, immune, fda, prevent;
- Topic 4—*Mistrust*: test, trial, mandate, child, year, old, force, kid, refuse;
- Topic 5—*Side effects*: effect, long, term, transmission, autoimmune, infection, spread;
- Topic 6—*Hiding relevant information* and *Side effects*: science, evidence, report, adverse, Pfizer, medical, research, article, reaction, safe, review.

Comparing the topics of the two periods, it can be observed that four of the topics, namely *Side effects*, *Existence of alternatives*, *Hiding relevant information*, and *Mistrust,* are the same. Additionally, in the Delta *cleaned* dataset, two more topics have been identified (*Scam* and *Freedom*). 

Considering the *Mistrust* reason, it has been observed that the speech has changed among the two periods: in the Delta *cleaned* dataset, the tweets were referring to not trusting the COVID-19 vaccine in general due to perceived insufficient testing, while in the Omicron *cleaned* dataset, the discourse was focused on the vaccination of children and the refusal to take the COVID-19 vaccine in the case of children.

It is interesting to notice that, as time has passed, some of the topics, such as the one related to the *Side effects*, have continued to be a part of the general discourse against COVID-19 vaccination. While it has been expected that a prolonged period of time since the start of the vaccination process would diminish the worries of the general population regarding the vaccine’s side effects, in fact, it appears that the opposite has occurred. 

Even in the case of the *Existence of alternatives*, it can be observed that some changes have occurred in the mentioned alternatives: while in the Delta *cleaned* dataset the alternatives included the wearing of masks and the presence of antibodies, in the Omicron *cleaned* dataset, masks were no longer mentioned, the alternative offered being only the natural immune system one possesses. 

The *Hiding relevant information* reason has continued to be a part of the discourse of the hesitant person in both periods. Looking closer at the tweets containing the words selected from each period with connection to *Hiding relevant information* reason, it has been observed that the tweets were referring to studies or persons who have spoken about the existence of evidence (e.g., studies, articles, review papers, reports) that supports the presence of harmful effects connected to the COVID-19 vaccine. 

It has been observed that the reasons related to one’s *Freedom* or the fact that the entire pandemic is a *Scam* have gradually disappeared from the hesitant discourse. 

#### 4.5.2. Hesitancy Reasons Dynamics

Furthermore, we thought it would be relevant to see how the reasons that have supported the hesitancy for COVID-19 vaccination have evolved over time during the entire duration of the pandemic. For this purpose, we have considered the results of two other studies on the COVID-19 vaccination topic [8,42], which have used the same keywords for the tweet extraction and which have assumed a 1-month period for analysis each. 

We have listed the COVID-19 vaccine hesitancy reasons from the two studies in Table 2 (marked with checkmarks), along with the reasons we have identified in the present study. The order of the columns under the “Event” column has been established based on the moment of time the studies have referred to, following a chronological approach. Additionally, the percentages of hesitant tweets in the other two studies [8,42] have also been included in Table 2.

Based on the information in Table 2, an increase in the percentage of *against* tweets can be observed from the start of the vaccination campaign (E1) to the occurrence of the Omicron variant (E4)—from 4.40% to 8.71% on *entire* datasets and from 6.78% to 11.33% for the *cleaned* datasets. This increase occurs against the background of a decrease in the number of COVID-19 vaccine hesitancy reasons—from seven reasons at the start of the vaccination campaign (E1) to four reasons in the Omicron period (E4)—which can lead to the conclusion that even though the number of reasons has been reduced, the remaining reasons have not been properly addressed through the vaccination information campaigns, which made their influence even more important in the vaccination decision process. 

Across all the periods, it can be noticed that the reasons for hesitancy have been related to the existence of *Side effects*. The *Side effects* have been seen at the start of the vaccination campaign (E1) in the form of some diseases, general health damage, sterilization, infertility, the occurrence of an autoimmune reaction that targets the placenta during pregnancy, the appearance of Bell’s Palsy disease, an increased risk of contracting the human immunodeficiency virus (HIV), an allergic/anaphylactic reaction, acquiring disabilities, brain bleeds or strokes, alteration of the deoxyribonucleic acid (DNA), and death [8]. They have then changed to the presence of blood clots, myocarditis, allergic reactions, and death during the E2, E3, and E4 periods. 

*Existence of alternatives* is another reason indicated in the hesitancy tweets, whose content has evolved from E1, where the possibility of using ivermectin, vitamin C, vitamin D, leronlimab, zinc, hydroxychloroquine, z-pak, or trusting the immune system [8] has been mentioned, to only trusting the immune system in the E2, E3, and E4 periods. 

*Hiding relevant information* has been a constant hesitancy reason across all the periods, as in each of the four periods (E1–E4) there have been studies, articles, or persons who have militantly supported the claim that there are hidden components (such as aluminum) in the vaccines, as the exact ingredients have not been disclosed to the population, or have mentioned that there are studies that prove the dramatic ill effects of the COVID-19 vaccine on people. The names of the mentioned persons and the content of the studies have changed during the four periods, but the discourse has stayed the same. 

The *Mistrust* reason has focused primarily on not trusting the use of the COVID-19 vaccine in the general population and has changed across time to mention the different groups who have been able to receive the vaccine (e.g., elderly, children, pregnant women). 

The *Scam* reason has been very prominent in E1, where issues related to population control and depopulation have been mentioned along with the fact that pharmaceutical companies earn billions as a result of the pandemic. Also, in many tweets posted in the E1, the vaccination of public persons who have been inoculated with the vaccine in public places or have been broadcast on television has been put under question, while an increased number of tweets in the E1 have suggested that the COVID-19 vaccine is in fact a placebo. During E2—as shown in this study—and E3, the discourse has continued to march on the fact that the vaccine is a means for the pharmaceutical companies to earn money. The other reasons for which the COVID-19 vaccine is a *Scam* seem to have been lost along the way. During E4, it can be observed that the reason related to the profits made by the pharmaceutical companies has lost interest and has not been mentioned among the hesitancy reasons. 

*Freedom* is one of the reasons related to each person’s choice of taking or not taking the vaccine. The reason appeared more frequently at the beginning of the vaccination campaign and during the occurrence of the Delta variant and has vanished in the last two periods (E3 and E4). 

*Inefficiency* has been mentioned mostly during the E1 period, when it was believed that the vaccine would protect only a small amount of the vaccinated population, and has not been used during the remainder of the periods (E2–E4).

## 5. Limitations

The present study is subject to limitations stemming from various decisions made during the modeling process. 

Firstly, the process of extracting tweets is notably sensitive to the chosen keywords, implying that utilizing a different set of keywords would yield an alternate dataset characterized by distinct attributes. 

Secondly, the study’s scope is confined by the chosen time period, thereby imposing a constraint on the extracted data solely within the designated timeframe. 

Thirdly, potential constraints arise from the classification procedure, as the classifier may not consistently grasp subtle linguistic nuances, such as the presence of irony in certain tweets. 

Furthermore, the linguistic composition of the tweets introduces an additional limitation, as this study exclusively incorporated tweets composed in English, as explicitly detailed in the paper.

Modifying any of these aforementioned components could conceivably result in slightly divergent outcomes.

## 6. Discussions and Conclusions

Gaining insights into people’s stances on COVID-19 vaccination holds pivotal significance in effective governance for managing the COVID-19 pandemic. Thus, the present study starts from the idea that harnessing extensive datasets extracted from social media, encompassing people’s sentiments regarding COVID-19 vaccination, enables the facile extraction and analysis of the impact of news or campaigns on shifts in attitudes toward such vaccination. To this extent, a large number of tweets (5,305,802 tweets) have been extracted for a one-month span subsequent to the announcement of Delta and Omicron variants (4 April 2021–3 May 2021 and 24 November 2021–23 December 2021, respectively) and analyzed through the use of a Transformer-based language model.

As a result of the analysis, the tweets dedicated to hesitancy when discussing the COVID-19 vaccination process have been selected. Based on the selected hesitancy tweets, the themes and underlying causes of COVID-19 vaccination hesitancy were investigated, revealing the most salient topics. Through a Latent Dirichlet Allocation approach, the main topics have been extracted, observing that a great deal of hesitancy lies in categories such as *Side effects*, *Existence of alternatives*, *Hiding relevant information*, *Mistrust*, *Scam*, or *Freedom* when the Delta variant occurs. As for the Omicron variant, the variety of COVID-19 hesitancy reasons has been diminished, with most of the reasons belonging to either the *Side effects*, *Existence of alternatives*, *Hiding relevant information*, or *Mistrust* categories. 

In comparison with the other two analyzed periods—namely, the initiation of the vaccination campaign and the introduction of the third booster dose—it has been observed that certain reasons for vaccine hesitancy have remained consistent over time (e.g., *Side effects*, *Existence of alternatives*, *Hiding relevant information*, and *Mistrust*), while other rationales have diminished (e.g., *Scam*, *Inefficiency*, and *Freedom*). 

Drawing from the hesitancy factors identified in this study (E2 and E4), substantiated by vaccine hesitancy drivers observed in two distinct timeframes (E1 and E3), a discernible trend emerges wherein certain rationales have gradually diminished in prevalence. This can be attributed to the efforts of vaccination campaigns, emerging research, personal experiences, and similar factors, resulting in their diminished representation within hesitant discourse on social media. Conversely, some reasons have seen a reduction in their prevalence due to partial resolution of associated concerns (for example, the discourse around favoring alternative pharmaceuticals for COVID-19 treatment instead of vaccination), leaving only a residual aspect to be addressed (such as exclusive reliance on natural immunity). Meanwhile, certain reasons have persisted in raising concerns over time (such as fears of the risk of death due to COVID-19 vaccines).

Considering other studies from the field dedicated to hesitancy but which have used a different method for data extraction (e.g., questionnaires applied to the population within a given region, such as a country, a state, or a city), it has been observed that some of the hesitancy reasons are the same. For example, *Mistrust* has been highlighted as one of the top-4 vaccine hesitancy reasons in a study conducted by Abdalla et al. [82] on the 1246 selected residents of Pune, while fear (which can be associated with either *Side effects*) has been listed as the top-1 vaccine hesitancy reason. Even more, the authors have reported high percentages for those doubting the vaccine efficiency and for the limited number of proper studies that prove the vaccine efficiency—reasons that can be associated with *Inefficiency* and *Hiding relevant information* [82]. Furthermore, Leung et al. [83] have shown that in a population set made up of respondents from Norway, the USA, the UK, and Australia, the main hesitancy reasons are represented by *Side effects* and *Mistrust*. Additionally, the authors have pointed out the hesitancy related to religious beliefs, which can be associated with the *Freedom* category in this study, showing once more that the reasons behind COVID-19 vaccine hesitancy have been merely the same across various territories [83]. Lack of trust in the vaccine, doubts related to the long-term effects, and refusal due to religious grounds—which can be associated with the *Mistrust*, *Side effects*, and *Freedom* categories identified in the current study—have been reported by Muluneh et al. [84] in a study on COVID-19 vaccine hesitancy in Ethiopia. Similar vaccine hesitancy reasons have been reported by Sun et al. [85] in the Chinese population, even though the grouping into the main categories has been slightly different than in the present study. Furthermore, Golebiowska et al. [86] in a study on the Polish population highlighted as reasons for hesitancy the fear of post-vaccination complications and safety (which is similar to the *Side effects* category in our study). 

Another important observation that can be highlighted from the present study is related to the percentage of tweets against COVID-19 vaccination, which has increased over time, raising questions for interested parties. As the pandemic has followed a downward trend, having been declared over by the WHO during May 2023 [87], this increase in the number of expressed hesitant opinions to COVID-19 vaccination might not bring immediate negative effects, but it is interesting to know and address the hesitancy reasons related to the vaccination process for the case in which a new COVID-19 variant might cause a new increase in the number of cases, or in the case of the occurrence of a new pandemic or other global calamity. Thus, using the proposed approach, the opinions of the general population can be easily measured along with the main reasons behind their decisions, and real-time information campaigns can be created and conducted to address their hesitancy. 

As some of the reasons for hesitancy remained over time, in the future, when similar pandemic situations might be encountered and a vaccination process is needed, the development of more tailored information campaigns that promptly address the underlying reasons for hesitancy should be considered.

## Figures and Tables

**Table 1:** Classification performance.

Classifier	Class	Precision	Recall	F-Score	Accuracy
SVM	*against*	0.81	0.64	0.71	71.97%
	*neutral*	0.71	0.83	0.77	
	*in favor*	0.66	0.69	0.68	
MNB	*against*	0.77	0.70	0.73	72.56%
	*neutral*	0.71	0.88	0.78	
	*in favor*	0.71	0.60	0.65	
RF	*against*	0.73	0.70	0.71	71.68%
	*neutral*	0.69	0.91	0.78	
	*in favor*	0.75	0.54	0.63	
BERT-base-cased	*against*	0.83	0.78	0.80	82.30%
	*neutral*	0.80	0.94	0.86	
	*in favor*	0.85	0.75	0.80	
BERT-base-uncased	*against*	0.90	0.84	0.87	85.84%
	*neutral*	0.81	0.96	0.88	
	*in favor*	0.88	0.78	0.83	
ALBERT-base-v2	*against*	0.82	0.88	0.85	86.13%
	*neutral*	0.86	0.94	0.90	
	*in favor*	0.91	0.77	0.83	
RoBERTa-base	*against*	0.89	0.99	0.94	95.57%
	*neutral*	1.00	0.97	0.99	
	*in favor*	0.99	0.90	0.94	

**Table 2:** Vaccine hesitancy reasons.

COVID-19 Vaccine Hesitancy Reasons	Event			
	E1: Start of the Vaccination Campaign [8]	E2: Delta (This Study)	E3: Third Vaccine Booster [42]	E4: Omicron (This Study)
	Period			
	8 December 2020–7 January 2021	4 April 2021–3 May 2021	12 July 2021–11 August 2021	24 November 2021–23 December 2021
	Percentage of *Against* Tweets (*Entire*/*Cleaned* Datasets)			
	4.40%/6.78%	4.47%/4.41%	4.89%/7.44%	8.71%/11.33%
Side effects	✔	✔	✔	✔
Existence of alternatives	✔	✔	✔	✔
Hiding relevant information	✔	✔	✔	✔
Mistrust	✔	✔	✔	✔
Scam	✔	✔	✔	
Inefficiency	✔			
Freedom	✔	✔		

## Data Availability

The dataset annotated during the current study is available at: https://github.com/liviucotfas/covid-19-vaccination-hesitancy-delta-omicron (accessed on 20 November 2022).
